# Influential factors of tuberculosis in mainland China based on MGWR model

**DOI:** 10.1371/journal.pone.0290978

**Published:** 2023-08-31

**Authors:** Zhipeng Ma, Hong Fan

**Affiliations:** State Key Laboratory of Information Engineering in Surveying, Mapping and Remote Sensing, Wuhan University, Wuhan, China; Shanxi University, CHINA

## Abstract

Tuberculosis (TB), as a respiratory infectious disease, has damaged public health globally for decades, and mainland China has always been an area with high incidence of TB. Since the outbreak of COVID-19, it has seriously occupied medical resources and affected medical treatment of TB patients. Therefore, the authenticity and reliability of TB data during this period have also been questioned by many researchers. In response to this situation, this paper excludes the data from 2019 to the present, and collects the data of TB incidence in mainland China and the data of 11 influencing factors from 2014 to 2018. Using spatial autocorrelation methods and multiscale geographically weighted regression (MGWR) model to study the temporal and spatial distribution of TB incidence in mainland China and the influence of selected influencing factors on TB incidence. The experimental results show that the distribution of TB patients in mainland China shows spatial aggregation and spatial heterogeneity during this period. And the R^2^ and the adjusted R^2^ of MGWR model are 0.932 and 0.910, which are significantly better than OLS model (0.466, 0.429) and GWR model (0.836, 0.797). The fitting accuracy indicators MAE, MSE and MAPE of MGWR model reached 5.802075, 110.865107 and 0.088215 respectively, which also show that the overall fitting effect is significantly better than OLS model (19.987574, 869.181549, 0.314281) and GWR model (10.508819, 267.176741, 0.169292). Therefore, this model is based on real and reliable TB data, which provides decision-making references for the prevention and control of TB in mainland China and other countries.

## 1. Introduction

According to a report by the World Health Organization (WHO, 2012), one-third of the world’s population, which is around two billion people infected with TB. As one of the top ten causes of death in the world, TB is a chronic infectious disease caused by Mycobacterium tuberculosis infection, which spreads mainly through respiratory transmission, and can be caused by recently infected patients or latently infected patients [1]. More than 90% of TB cases and deaths come from developing countries. In 2021, the estimated incidence of TB in China ranked third after India and Indonesia, so TB was listed as a Class-B respiratory infectious disease in China. In recent years, due to Chinese government’s increased prevention and control efforts, the number of patients in China has been decreasing year by year. In addition, due to the large population base and the huge latent infection population in China, the number of patients in some areas has rebounded and increased in recent years. Therefore, TB is still one of the main infectious diseases that endangers the health of Chinese residents. Since the widespread spread of COVID-19, as COVID-19 has formed severe occupation of medical resources and disrupted the health care system [2], TB statistics have dropped significantly. Although some researchers believe that the temporary immunosuppressive effects and the corticosteroids used to treat COVID-19 have played a direct role in immunosuppression of TB [3], the authenticity and reliability of TB data are still questioned by many researchers.

In recent years, with the development of artificial intelligence data analysis and the improvement of data integrity, more and more researchers around the world have conducted varying degrees of research on the incidence distribution and pathogenic factors of TB. With the continuous development of the research level, the discussion of factors affecting TB incidence is no longer limited to the source of infection, the route of transmission, and the susceptible population. There is evidence to prove that meteorological factors and exposure to air pollutants have a certain impact on TB [4]. Due to transmission through the respiratory tract, TB has obvious seasonal peaks, with a larger peak in late spring (April) and a smaller peak in early autumn [5], and some studies have confirmed that long-term exposure to SO_2_ [6] or occupational inhalation of silica dust [7] will increase the risk of men suffering from TB. Meanwhile, meteorological factors such as temperature and relative humidity may influence the risk of TB by altering the temporal and spatial distribution of air pollutants [8], resulting in spatial heterogeneity in the distribution of TB incidence in various regions of the world. TB is known as a disease reflecting socio-economic and environmental conditions [9], and regional differences in socio-economic are also one of the reasons for its spatial heterogeneity [10]. Different researchers use different models to analyze local TB incidence and socio-economic factors, and all conclude that there is a significant curvilinear relationship with socio-economic status [10, 11], which further verify the importance of socio-economic on the spread of TB. But the incidence of TB is affected by many factors, for example, TB incidence and corresponding mortality rates in S. Korea are unusual and unique compared to other economically developed countries [11]. At the same time, some researchers conduct a multilevel analysis of self-reported TB on the representative sample of South Africa, and find that TB is associated with cigarette smoking, alcohol consumption, low body mass index, lower level of personal education, lower household wealth and unemployment [12]. And other researchers conduct a retrospective analysis on routinely collected TB data in Kenya, and conclude that TB is not only related to age and gender, but also differences in nationality and job can lead to changes in the growth of TB [13].

The current incidence of TB in China also shows spatial heterogeneity [14], so systematic investigations of social and environmental factors influencing TB are necessary for the prevention and control of the disease [15]. In recent years, researchers have successively carried out research on areas with high incidence of TB in economically underdeveloped areas in western China. Some researchers applied Kulldorff ’s spatial-temporal scanning statistics to identify the temporal and spatial clusters of county-level TB prevalence in Yunnan, detected aggregated time interval and regions for TB at county-level of Yunnan Province, and found similarity prevalence patterns in the borders of China and the Great Mekong Subregion (GMS) region [16]. The spatial clustering of TB among students in Nanning, mainly located in the urban center and its surrounding areas, and the clustering gradually decreased from the urban center to the surrounding areas [17]. Meanwhile, some studies have shown that air quality and economic level have hysteresis with different lag time [15], and find that using ’’proportion of minorities’’ as a predictor may help to guide TB control programs and targeting interventions [18].

Since most infectious diseases have the characteristics of spatial aggregation and spatial heterogeneity, traditional regression models often have problems such as insufficient discussion of spatial distribution characteristics when dealing with such problems, resulting in unsatisfactory analysis results. At present, most research methods of TB are relatively single, mainly including Spatial Lag Model [1, 18], Spatial Error Model [18, 19], Multivariate Poisson Regression Model [20], etc. And the analysis of TB needs to consider spatial distribution characteristics [21, 22], so a single analysis model can’t accurately analyze such problems. Therefore, this paper takes Moran’s I as the analysis index to carry out the research on global and local spatial autocorrelation. On this basis, MGWR model, which is often applied to urban housing land prices [23, 24], economic development [25], medical and health field [26], but not often used in the exploration of disease influencing factors, is selected to analyze the influencing factors of TB and its influence degree. By analyzing the spatial distribution of TB and reasonably inferring the potential relationship between TB and other influencing factors, this paper provides decision-making references for the prevention and control of TB in the future.

## 2. Materials and methods

### 2.1 Research area

**Fig 1:**
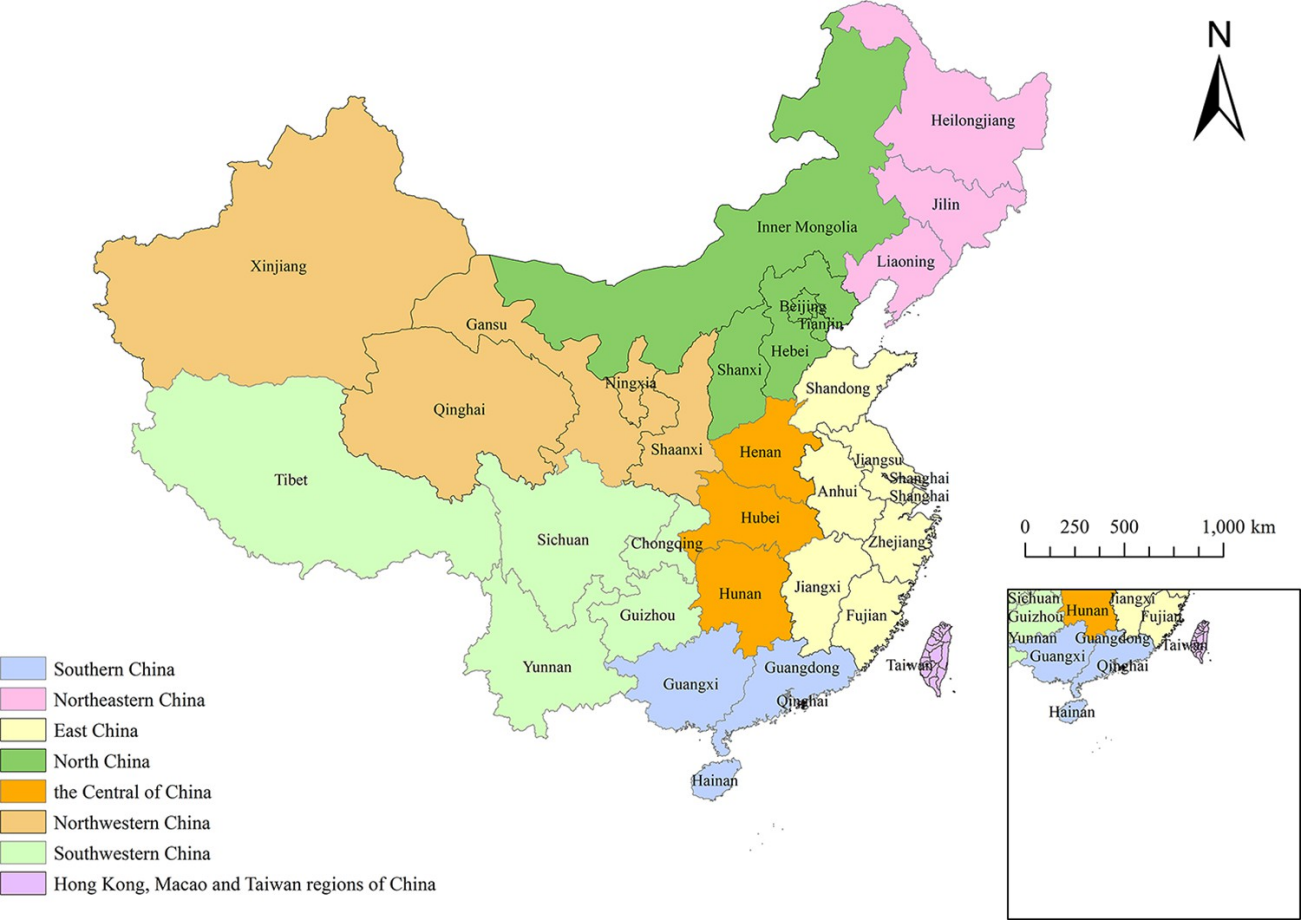
Locations of study areas in mainland China.

China, a country of East Asia with a land area of about 9.6 million square kilometers, has 34 provincial-level administrative regions, including 23 provinces, 5 autonomous regions, 4 municipalities and 2 special administrative regions. According to data released by National Bureau of Statistics of China on January 17, 2020, the total population of mainland China exceeded 1.4 billion, ranking first in the world. In order to formulate effective prevention and control measures to reduce the serious harm caused by TB, it is urgent to select real and reliable TB data and find out the factors that affect the spread of TB. The research area is 31 provinces in mainland China (municipalities and autonomous regions, excluding Hong Kong, Macao and Taiwan), as shown in Fig 1. The map comes from Natural Earth (http://www.naturalearthdata.com/).

### 2.2 Data collection

**Fig 2:**
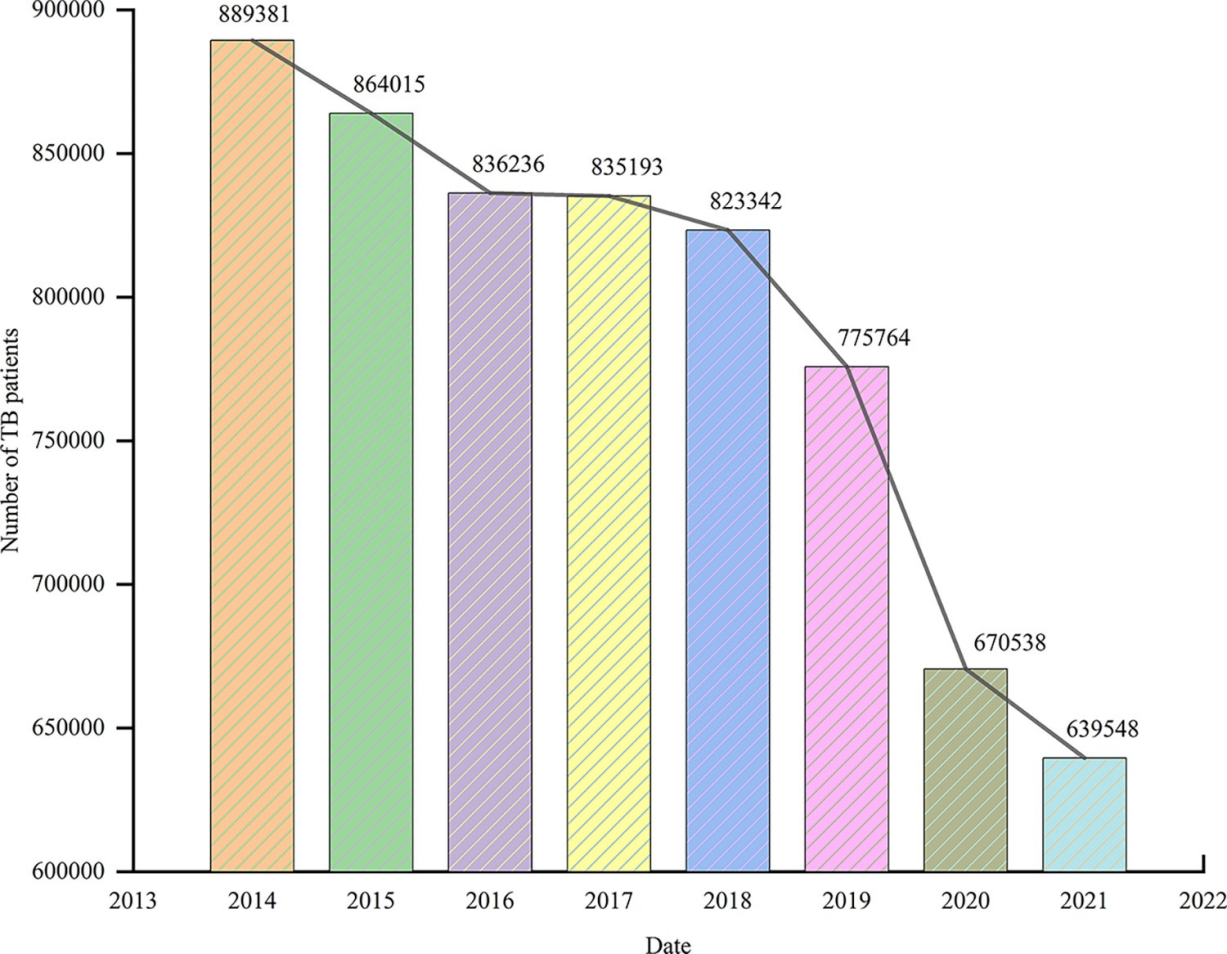
Trends in the number of TB patients in mainland China from 2014 to 2021.

Since the outbreak of COVID-19, the number of TB cases in mainland China has dropped significantly, as shown in Fig 2. One of the reasons is that mainland China was affected by COVID-19, and most of medical resources were occupied, while the control of TB was neglected. Another reason is some patients failed to go to the hospital in time, resulting in deviations in the statistics. Therefore, in order to ensure the authenticity and reliability of TB research data, this study excludes the data from 2019 to the present, analyzes the situation in the five years before the outbreak of COVID-19, and collects relevant data from different official websites. TB data of each province and city in China from January 2014 to December 2018 comes from Public Health Science Data Center (https://www.phsciencedata.cn/).

Because TB is an infectious disease that spreads through the respiratory tract, air quality will directly affect the incidence of TB [14, 27, 28]. In recent years, the awareness of environmental protection in various countries has continuously improved, and the importance of air quality has been repeatedly emphasized. PM_2.5_, SO_2_ and NO_2_, as the main pollutants for judging the quality of air, can directly enter the lungs through respiratory tract and affect human health [55, 56, 58]. In severe cases, they may cause alveolar atrophy, pulmonary edema and other problems [59], which leads to the onset of TB. Therefore, this study selects the data of PM_2.5_, SO_2_ and NO_2_ from 2014 to 2018 as the factors affecting the relationship between air quality and TB.

However, TB is a disease that reflects socio-economic and environmental conditions [9], and the level of social life is also one of the important factors affecting TB. Per capita consumption expenditure, Average number of clinic visits, Per capita healthcare consumption expenditure, Per capita GDP and Passenger traffic volume are important factors to evaluate the level of social life in a region, which largely reflects the local economic level and development level. Due to the differences in social life in different regions, the investment in healthcare costs during the early prevention period is also different, which has also led to a significant decline in the incidence of TB in some regions with higher social living standards [11, 14, 18, 29]. Therefore, this study selects data such as Per capita consumption expenditure, Average number of clinic visits, Per capita healthcare consumption expenditure, Per capita GDP and Passenger traffic volume from 2014 to 2018 as the influencing factors of social life and TB.

Moreover, TB is an infectious disease, and medical level will directly affect TB [14, 30, 31]. Number of beds in medical institutions, Number of health technicians per thousand population, and Number of medical institutions all reflect the medical level in a region, which in turn reflects the ability to detect and treat patients with TB in time, thereby reducing the spread of TB. Therefore, this study selects data such as Number of beds in medical institutions, Number of health technicians per thousand population, and Number of medical institutions from 2014 to 2018 as the factors influencing the relationship between medical level and TB, see Table 1.

**Table 1:** Variable list of influencing factors.

Dimension	Indicator	Source	Description
**air quality**	PM_2.5_	China Air Quality Online Monitoring and Analysis Platform (https://www.aqistudy.cn/)	Annual average concentration of PM_2.5_ in mainland China from 2014 to 2018
	SO_2_	China Air Quality Online Monitoring and Analysis Platform (https://www.aqistudy.cn/)	Annual average concentration of SO_2_ in mainland China from 2014 to 2018
	NO_2_	China Air Quality Online Monitoring and Analysis Platform (https://www.aqistudy.cn/)	Annual average concentration of NO_2_ in mainland China from 2014 to 2018
**social life**	Per capita consumption expenditure	China Statistical Yearbook (http://www.stats.gov.cn/tjsj./ndsj)	Annual data from 2014 to 2018
	Average number of clinic visits	China Statistical Yearbook (http://www.stats.gov.cn/tjsj./ndsj)	Annual data from 2014 to 2018
	Per capita healthcare consumption expenditure	China Statistical Yearbook (http://www.stats.gov.cn/tjsj./ndsj)	Annual data from 2014 to 2018
	Per capita GDP	China Statistical Yearbook (http://www.stats.gov.cn/tjsj./ndsj)	Annual data from 2014 to 2018
	Passenger traffic volume	China Statistical Yearbook (http://www.stats.gov.cn/tjsj./ndsj)	Annual data from 2014 to 2018, including railways, water transport and civil aviation
**medical level**	Number of beds in medical institutions	China Statistical Yearbook (http://www.stats.gov.cn/tjsj./ndsj)	Annual data from 2014 to 2018, including hospitals, primary health institutions and professional public health institutions
	Number of health technicians per thousand population	China Statistical Yearbook (http://www.stats.gov.cn/tjsj./ndsj)	Annual data from 2014 to 2018, including urban and rural
	Number of medical institutions	China Statistical Yearbook(http://www.stats.gov.cn/tjsj./ndsj)	Annual data from 2014 to 2018, includes hospitals, primary health institutions and professional public health institutions

### 2.3 Technical route

**Fig 3:**
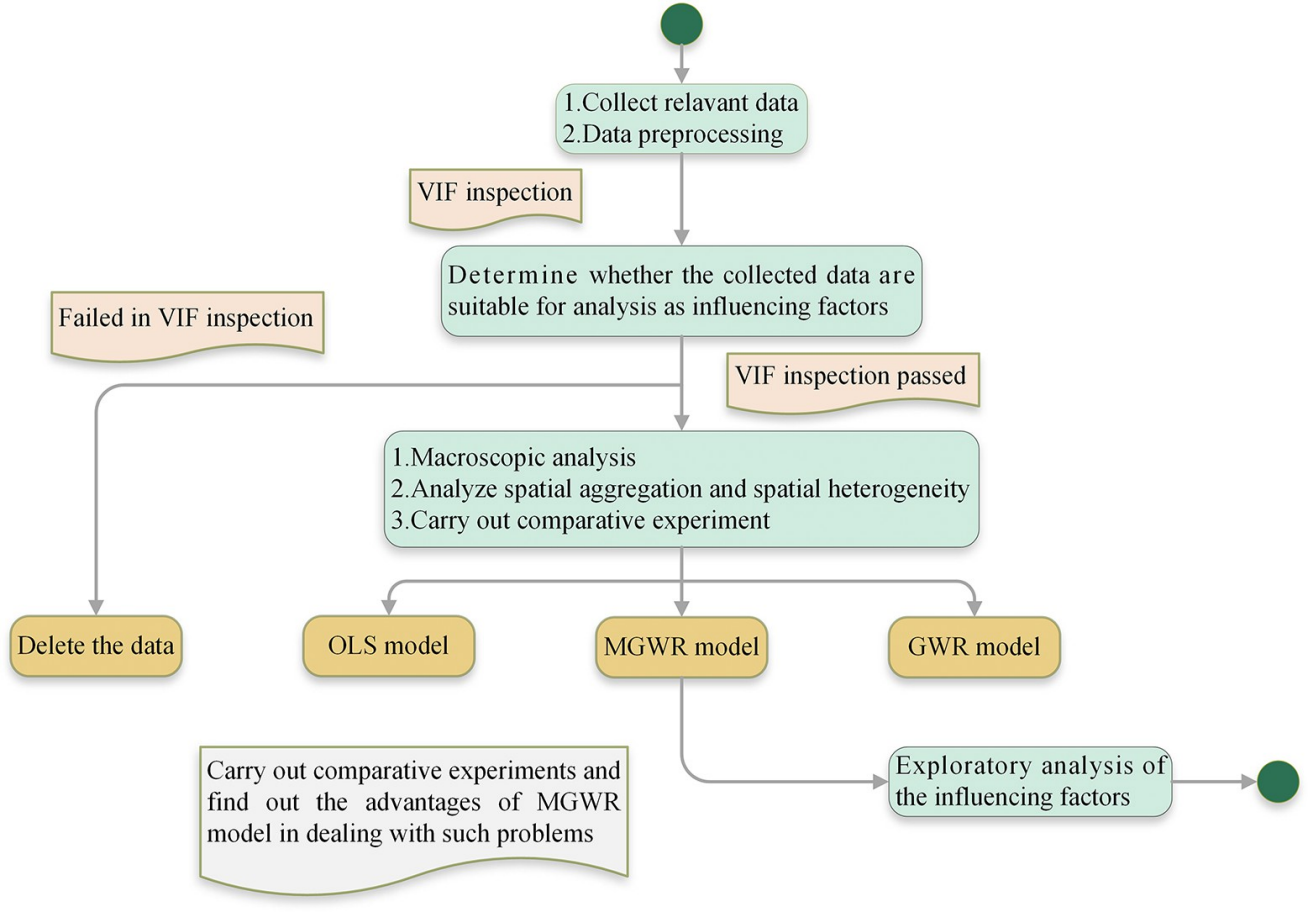
Main technical route of this study.

The technical route of this study is shown in Fig 3, and the important research contents and steps are briefly introduced as follows:

1. Collect relevant data. Including TB data in mainland China from 2014 to 2018, 11 influencing factors data, and map resource data.
2. Preprocess data. Including the VIF test on 11 influencing factors to judge whether they are suitable for analysis as impact factors, and linear transformation of each data to facilitate model calculation.
3. Macroscopic analysis of the distribution of TB incidence in mainland China from 2014 to 2018.
4. Analyze the spatial distribution characteristics of TB by spatial autocorrelation methods and analyze spatial heterogeneity of TB by MGWR model.
5. Conduct comparative experiments. Verify the advantages of MGWR model in dealing with such problems through the comparison of OLS model, GWR model and MGWR model.
6. Carry out exploratory analysis of the influencing factors. Based on the coefficients and significance obtained from the analysis of MGWR model, targeted suggestions are put forward for the prevention and control of TB.

#### 2.3.1 Spatial autocorrelation

In order to study the temporal and spatial distribution characteristics of TB, this study uses spatial autocorrelations method to explore the spatial distribution and spatial aggregation of TB incidence in mainland China in the five years before the outbreak of COVID-19, namely from 2014 to 2018. It can help us provide scientific decision-making support for the precise prevention and control of TB, taking mainland China as an example, when the authenticity and reliability of TB data has been reduced after the outbreak of COVID-19. And spatial autocorrelation refers to the potential dependence of some factors in a region, which can be roughly divided into two categories in terms of function: one is global spatial autocorrelation and the other is local spatial autocorrelation [32–35]. The global spatial autocorrelation method is used to test whether the whole region has correlation. The most widely used index is the global Moran’s I index, which is often used to indicate the degree of correlation between each region and other regions, it is defined as follows:

$$I=\frac{n\sum_{i=1}^{n}\sum_{j=1}^{n}w_{ij}(x_{i}-\bar{x})(x_{j}-\bar{x})}{(\sum_{i=1}^{n}\sum_{j=1}^{n}w_{ij})\sum_{i=1}^{n}{\left(x_{i}-\bar{x}\right)}^{2}}$$ (1)

Where *n* represents the number of statistical provinces and cities, x_i_ and x_j_ represent the annual incidence of TB in provinces *i* and *j*, respectively, $\bar{x}$ represents the annual average incidence of 31 provinces and cities, w_ij_ is the spatial weight matrix, which is defined as follows:

$$w_{ij}=\{\begin{array}{l} 1,\;i,j\;provinces\;and\;cities\;are\;adjacent\;or\;have\;a\;common\;edge\\ 0,\;i,j\;provinces\;and\;cities\;aren\prime t\;adjacent\;or\;have\;no\;common\;edge \end{array}$$ (2)

The value range of Moran’s I index is −1<*I*<1. When *I*>0, it indicates that the study area is clustered between high-incidence areas and high-incidence areas, low-incidence areas and low-incidence areas, namely positive correlations. When *I*<0, it indicates that the study area shows a mixed aggregation distribution of high-incidence areas and low-incidence areas, namely negative correlations. When *I* = 0, it indicates that the study area is randomly aggregated in high-incidence areas and low-incidence areas, namely no correlations.

The local spatial autocorrelation method is used to test whether the region have local aggregation when the whole region has correlation. This paper also uses the local Moran’s I index for analysis, and its definition is shown in Formula 3, where the definitions of *n*, *x*_*i*_, *x*_*j*_ and *w*_*ij*_ are the same as those defined in the global spatial autocorrelation method.


$$I_{i}=\frac{n(x_{i}-\bar{x})}{\sum_{i=1}^{n}{\left(x_{i}-\bar{x}\right)}^{2}}\sum_{j=1,j\neq i}^{n}w_{ij}(x_{j}-\bar{x})$$ (3)

Z test is often applied to the detection of the difference in the average value of large samples. It compares the Z score of the difference between the two averages with the specified theoretical Z score to determine whether the difference between the two averages is significant, and the relationship between the Z score and the significance of the difference is shown in Table 2. Through comparing the positive and negative values of Moran’s I with the Z test value, four spatial distributions can be obtained: high-high (H-H) aggregation, high-low (H-L) aggregation, low-high (L-H) aggregation, low-low (L-L) aggregation [36].

**Table 2:** Relationship between Z score and significance of difference.

Z score	P value	Significance of difference
> 2.58	< 0.01	very significant
> 1.96	< 0.05	significant
≤ 1.96	≥ 0.05	not significant

#### 2.3.2 Multiscale Geographically Weighted Regression (MGWR) model

MGWR model is one of the important models for analyzing spatial heterogeneity, which is often used in urban housing land prices, economic development, medical and health fields, but not often used in the exploration of disease influencing factors. MGWR model is an optimization model of GWR. On the one hand, it introduces the location characteristics of each sample point in space, and takes the distance between sample points as an important factor to define the weight, which can effectively reveal the spatial imbalance distribution. On the other hand, MGWR model further divides the research variables into global variables without significant spatial heterogeneity and local variables with significant spatial heterogeneity [37–41], and sets different bandwidths for each research variable, which can better explain the spatial effects of the research variables. Therefore, this paper uses MGWR model to explore the influencing factors of TB.

In order to better introduce MGWR model, this paper first introduces OLS model and GWR model. OLS model is often used to deal with the problem that a dependent variable is affected by multiple independent variables. The most common analytical model is Multiple Linear Regression Model, where *y* is the dependent variable, and *x*_1_、*x*_2_…*x*_*i*_ are independent variables, and they are linearly related. This study adopts this model for analysis, expressed as follows:

$$y=\beta_{0}+\beta_{i}x_{i}+\epsilon_{i}$$ (4)

Where β_0_ is the constant, β_*i*_ is the regression coefficient of independent variable *i*, *x*_*i*_ is the actual value of independent variable *i*, and ϵ_*i*_ is the random error of independent variable *i*.

The difference between GWR model and ordinary linear regression model is that the spatial location information is added, and the regression coefficients of each area are analyzed separately [42–45]. First, the expression of GWR model is given as follows:

$$y_{i}=\beta_{0}(u_{i},v_{i})+\sum_{k=1}^{k}\beta_{k}(u_{i},v_{i})x_{ik}+\epsilon_{i}$$ (5)

Where *y*_*i*_ is the predicted value of observation point *i*, β_0_(*u*_*i*_, *v*_*i*_) is the constant whose position (*u*_*i*_, *v*_*i*_) is the longitude and latitude of observation point *i*, β_*k*_(*u*_*i*_, *v*_*i*_) is the regression coefficient of observation point *i* to independent variable *k*, *x*_*ik*_ is the actual value of observation point *i* to independent variable *k*, ϵ_*i*_ is the random error of observation point *i*.

The spatial weight matrix *W*(*u*_*i*_, *v*_*i*_) is the core of GWR model. The selection of the appropriate weight function is crucial to the accuracy of regression model, and the commonly used weight functions are Gaussian function and Bi-square function [46]. Among them, Gaussian function discusses all samples in the spatial scope, while Bi-square function only discusses the samples within the bandwidth range, which can avoid discussing some samples that have no impact on the regression coefficients. Therefore, when using GWR model, Bi-square function is often used as its spatial weight matrix, and it is expressed as follows:

$$w_{ij}=\{\begin{array}{l} {\left(1-\frac{d_{ij}^{2}}{b^{2}}\right)}^{2},d_{ij}<b\\ \;0\;,d_{ij}\geq b \end{array}$$ (6)

Where *w*_*ij*_ is the weight factor of fitting point *i* and sample point *j*, *d*_*ij*_ is the distance of fitting point *i* and sample point *j*, and *b* is the bandwidth which represents the degree of non-negative attenuation between weight and distance.

When the bandwidth is selected, fixed function and adaptive function are often used. Fixed function is suitable for uniform distribution of sample points, and each sample point uses same bandwidth to analyze. Adaptive function is suitable for discrete distribution of sample points, and each sample point continuously adjusts the bandwidth *b* to make the model optimal [47]. Since the distribution of TB incidence is discrete, the adaptive function is used to analyze it.

MGWR model avoids the over-analysis of some independent variables in the GWR model, and divides the *k* independent variables in Formula (5) into *p* global variables without significant spatial heterogeneity and *k*−*p* local variables with significant spatial heterogeneity. It can be shown as:

$$y_{i}=\beta_{0}(u_{i},v_{i})+\sum_{k=1}^{p}\beta_{k}(u_{i},v_{i})x_{ik}+\sum_{k=p}^{k}\beta_{k}(u_{i},v_{i})x_{ik}+\epsilon_{i}$$ (7)

MGWR model can be regarded as a mixed model of GWR model and general linear regression model, which can judge spatial heterogeneity of each independent variable, speed up the calculation convergence speed and improve the fitting degree.

#### 2.3.3 Primary selection of influencing factors

This paper builds a regression model based on the selected 11 influencing factors. In the establishment of MGWR model, some variables may have a certain degree of correlation with other variables, so these variables are defined as having multicollinearity. In order to ensure the rationality of statistical analysis, factors with multicollinearity should be eliminated. In this paper, variance inflation factor (VIF) test [48] is used to help eliminate collinearity problems, and some influencing factors with VIF > 10 can be considered to have multicollinearity.

We conducted the VIF test on the selected variables in Table 1, including air quality, social life, and medical level. Table 3 shows that the VIF value of Per capita consumption expenditure is greater than 10, and this factor is considered to have multicollinearity, so it is no longer participate in subsequent modeling. The VIF of the other 10 influencing factors are all less than 10, and it can be considered that these influencing factors have no multicollinearity, so these factors are retained to continue to participate in subsequent modeling.

**Table 3:** VIF test of influencing factors.

Dimension	Indicator	VIF
**air quality**	PM_2.5_	3.540
	SO_2_	1.916
	NO_2_	2.856
**social life**	Per capita consumption expenditure	16.097
	Average number of clinic visits	4.948
	Per capita healthcare consumption expenditure	5.009
	Per capita GDP	8.082
	Passenger traffic volume	3.443
**medical level**	Number of beds in medical institutions	8.790
	Number of health technicians per thousand population	2.501
	Number of medical institutions	6.612

#### 2.3.4 Comparative experiment

In this paper, OLS model and GWR model are selected to carry out comparative experiments to verify the performance of MGWR model. The OLS model is realized by SPSS24.0 software, the GWR model is realized by GWR4 software, and the MGWR model is realized by MGWR2.2 software. Among them, many criteria are used to judge the fitting effect of statistical model. The most common one is AIC (Akaike Information Criterion), which is used as a standard to measure the fitting effect of statistical model and is defined as follows:

$$AIC=2n+nln(\frac{SSR}{n})$$ (8)

$$SSR=\sum_{i=1}^{n}{\left(x-\bar{x}\right)}^{2}$$ (9)

Where *n* is the number of statistical sample points, *x* is the predicted data of observation point, $\bar{x}$ is the actual value of observation point, and SSR is residual sum of squares. Among them, the lower the SSR and AIC, the smaller the prediction deviation of the model and the better the fitting effect. Meanwhile, use Mean Absolute Error MAE, Mean Square Error MSE and Mean Absolute Percentage Error MAPE to compare the error between the predicted value and the actual value, which are defined as follows:

$$MAE=\frac{1}{n}\sum_{i=1}^{n}\left|\hat{y_{i}}-y_{i}\right|$$ (10)

$$MSE=\frac{1}{n}\sum_{i=1}^{n}{\left(\hat{y_{i}}-y_{i}\right)}^{2}$$ (11)

$$MSE=\frac{1}{n}\sum_{i=1}^{n}\frac{\left|\hat{y_{i}}-y_{i}\right|}{y_{i}}$$ (12)

Where $\hat{y_{i}}$ is the predicted value of observation point *i*, and *y*_*i*_ is the actual value of observation point *i*.

## 3. Experiments and results

### 3.1 Macroscopic analysis and spatial autocorrelation experiment of TB

**Fig 4:**
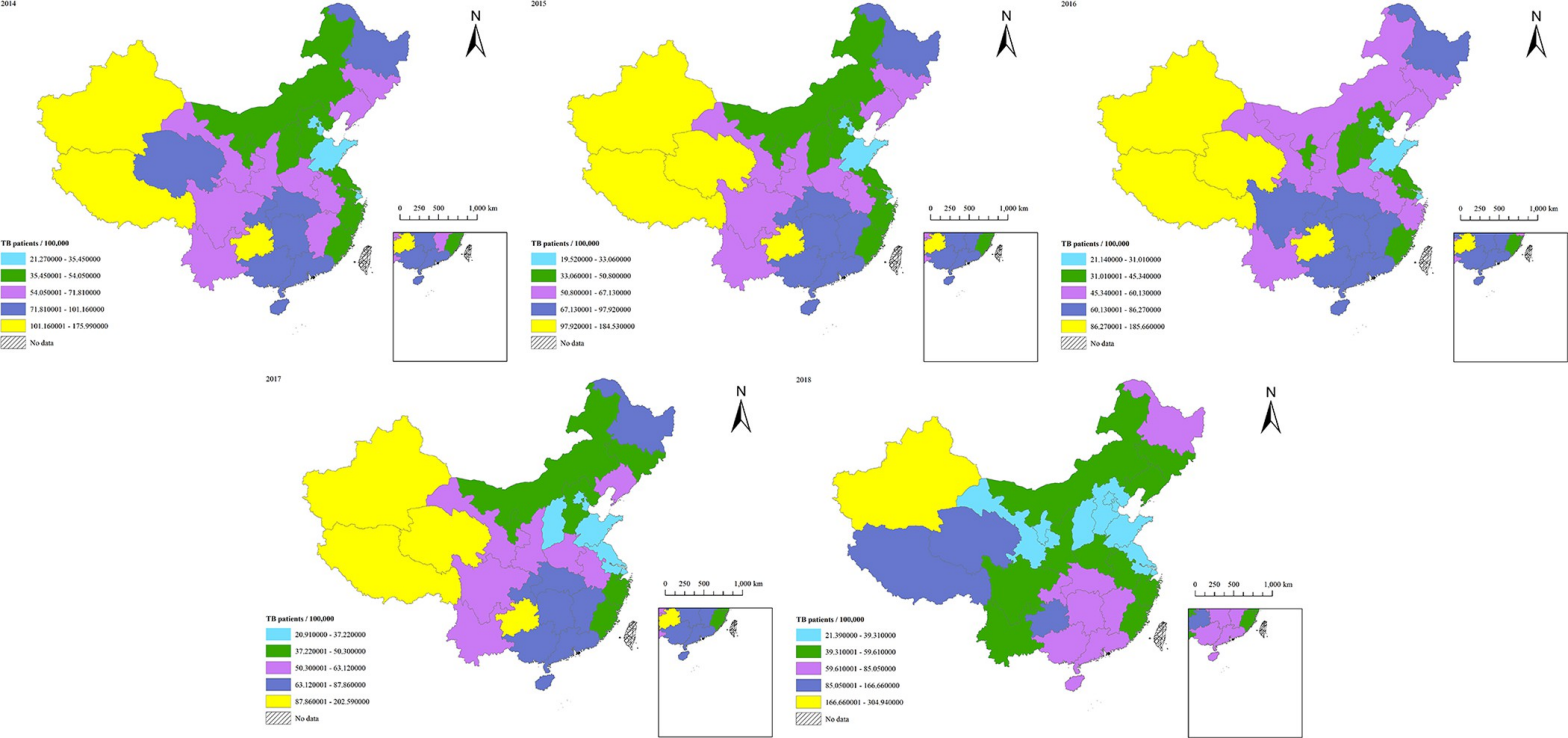
Distribution of TB incidence in mainland China from 2014 to 2018.

The spatial distribution of TB incidence in mainland China from 2014 to 2018 is shown in Fig 4. The areas with the highest annual average incidence of TB were Xinjiang (211.81/100,000), Tibet (153/100,000), Guizhou (125.89/100,000), Qinghai (125.69/100,000) and the areas with the lowest annual average incidence of TB were Tianjin (20.85/100,000), Shanghai (27.25/100,000), Shandong (31.49/100,000) and Beijing (32.09/100,000). The overall incidence showed a downward trend from 2014 to 2018, but the incidence in Qinghai, Tibet and Xinjiang increased from 101.16/100,000, 147.99/100,000, 175.99/100,000 to 140.33/100,000, 166.66/100,000, 304.94/100,000, respectively.

**Fig 5:**
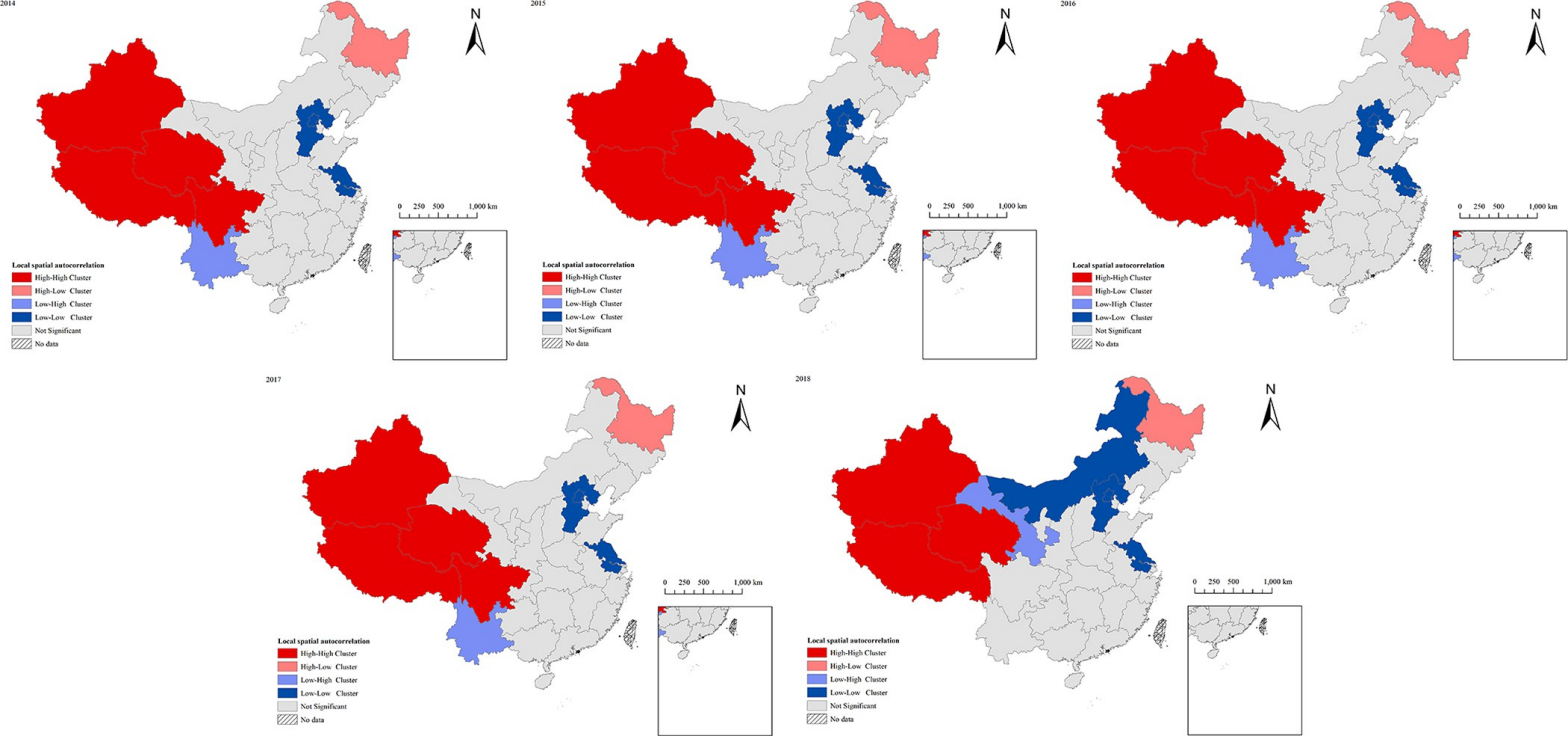
Distribution of local clusters of TB in mainland China from 2014 to 2018.

The data of TB incidence in mainland China are associated with the vector map of mainland China through ArcGIS10.8, and the results of global spatial autocorrelation are shown in Table 4. The values of Moran’s I from 2014 to 2018 are all greater than 0, and the P values are all less than 0.01, indicating that TB incidence has spatial aggregation in the study area. Local spatial autocorrelation analysis shows that there are four spatial distributions of TB in mainland China, as shown in Fig 5. The H-H clusters from 2014 to 2017 are concentrated in Xinjiang, Tibet, Qinghai and Sichuan, the H-L clusters are concentrated in Heilongjiang, the L-H clusters are concentrated in Yunnan, and the L-L clusters are concentrated in Beijing, Hebei and Jiangsu. Moreover, the H-H clusters in 2018 are concentrated in Xinjiang, Tibet and Qinghai, the H-L clusters are still concentrated in Heilongjiang, the L-H clusters are concentrated in Gansu, and the L-L clusters are concentrated in Beijing, Hebei, Jiangsu and Inner Mongolia. It indicates the presence of local clusters of TB within the study area.

**Table 4:** Spatial aggregation analysis results of TB in mainland China from 2014 to 2018.

year	Moran’s I	Z score	P value
**2014**	0.443014	4.235262	< 0.01
**2015**	0.454716	4.341855	< 0.01
**2016**	0.486561	4.662733	< 0.01
**2017**	0.499934	4.887245	< 0.01
**2018**	0.384110	4.519545	< 0.01

### 3.2 Experiments and comparative experiments of MGWR analysis

The selected influencing factors are analyzed through OLS model, and the analysis results are shown in Table 5. At this point, Formula 4 is transformed into:

$$y=125.19818+0.44310x_{1}+1.01356x_{2}+0.03326x_{3}-5.74165x_{4}-0.02633x_{5}-0.00057x_{6}-0.00008x_{7}+0.29768x_{8}+10.07590x_{9}-0.00072x_{10}$$

Where *x*_1_ represents the actual value of PM_2.5_, *x*_2_ represents the actual value of SO_2_, *x*_3_ represents the actual value of NO_2_, *x*_4_ represents the actual value of Average number of clinic visits, *x*_5_ represents the actual value of Per capita healthcare consumption expenditure, and *x*_6_ represents the actual value of Per capita GDP, *x*_7_ represents the actual value of Passenger traffic volume, *x*_8_ represents the actual value of Number of beds in medical institutions, *x*_9_ represents the actual value of Number of health technicians per thousand population, *x*_10_ represents the actual value of Number of medical institutions.

**Table 5:** Parameters of OLS model.

Dimension	Indicator	Coefficient
**air quality**	Constant	125.19818
	PM_2.5_	0.44310
	SO_2_	1.01356
	NO_2_	0.3326
**social life**	Average number of clinic visits	-5.74165
	Per capita healthcare consumption expenditure	-0.02633
	Per capita GDP	-0.00057
	Passenger traffic volume	-0.00008
**medical level**	Number of beds in medical institutions	0.29768
	Number of health technicians per thousand population	10.07590
	Number of medical institutions	-0.00072

Compared with OLS model, the core point of GWR model is to add spatial location information and introduce weight matrix, and the results are shown in Table 6. For specific parameters, see S4 Dataset.

**Table 6:** Parameters of GWR model.

Dimension	Indicator	Mean	Standard Deviation
**air quality**	Constant	-0.287	0.152
	PM_2.5_	0.023	0.303
	SO_2_	0.207	0.318
	NO_2_	-0.068	0.215
**social life**	Average number of clinic visits	-0.319	0.252
	Per capita healthcare consumption expenditure	-0.083	0.279
	Per capita GDP	0.130	0.387
	Passenger traffic volume	-0.068	0.295
**medical level**	Number of beds in medical institutions	0.175	0.225
	Number of health technicians per thousand population	-0.054	0.355
	Number of medical institutions	-0.305	0.489

The bandwidth of MGWR model reflects the effect of the influencing factors on the model. The bandwidth is larger, the spatial heterogeneity of the influencing factors is less obvious, and vice versa [49]. As shown in Table 7, the bandwidth of SO_2_ in air quality factors is 46, and the spatial heterogeneity is not obvious. However, the bandwidths of PM_2.5_ and NO_2_ are 151 and 137, respectively, which are basically equal to the total number of samples, so they are global variables. Among the social life factors, the bandwidths of Average number of clinic visits, Per capita healthcare consumption expenditure, Per capita GDP and Passenger traffic volume are 46, 46, 67 and 46, respectively, with little spatial heterogeneity. In the medical level factors, the bandwidths of Number of health technicians per thousand population and Number of medical institutions are 56 and 56, respectively, which shows that the spatial heterogeneity is not obvious. However, the bandwidth of Number of beds in medical institutions is 122, which is almost equal to the total sample size, so it is a global variable. According to the t-test table, the adjusted t-values are all greater than 1.812, which indicates that the selected impact factors are significantly credible at the 95% confidence level.

**Table 7:** Parameters of MGWR model.

Dimension	Indicator	bandwidth	Adjust t value
**air quality**	Constant	46	2.492
	PM_2.5_	151	2.029
	SO_2_	46	2.605
	NO_2_	137	2.149
**social life**	Average number of clinic visits	46	2.564
	Per capita healthcare consumption expenditure	46	2.659
	Per capita GDP	67	2.363
	Passenger traffic volume	46	2.574
**medical level**	Number of beds in medical institutions	122	2.100
	Number of health technicians per thousand population	56	2.528
	Number of medical institutions	56	2.410

**Fig 6:**
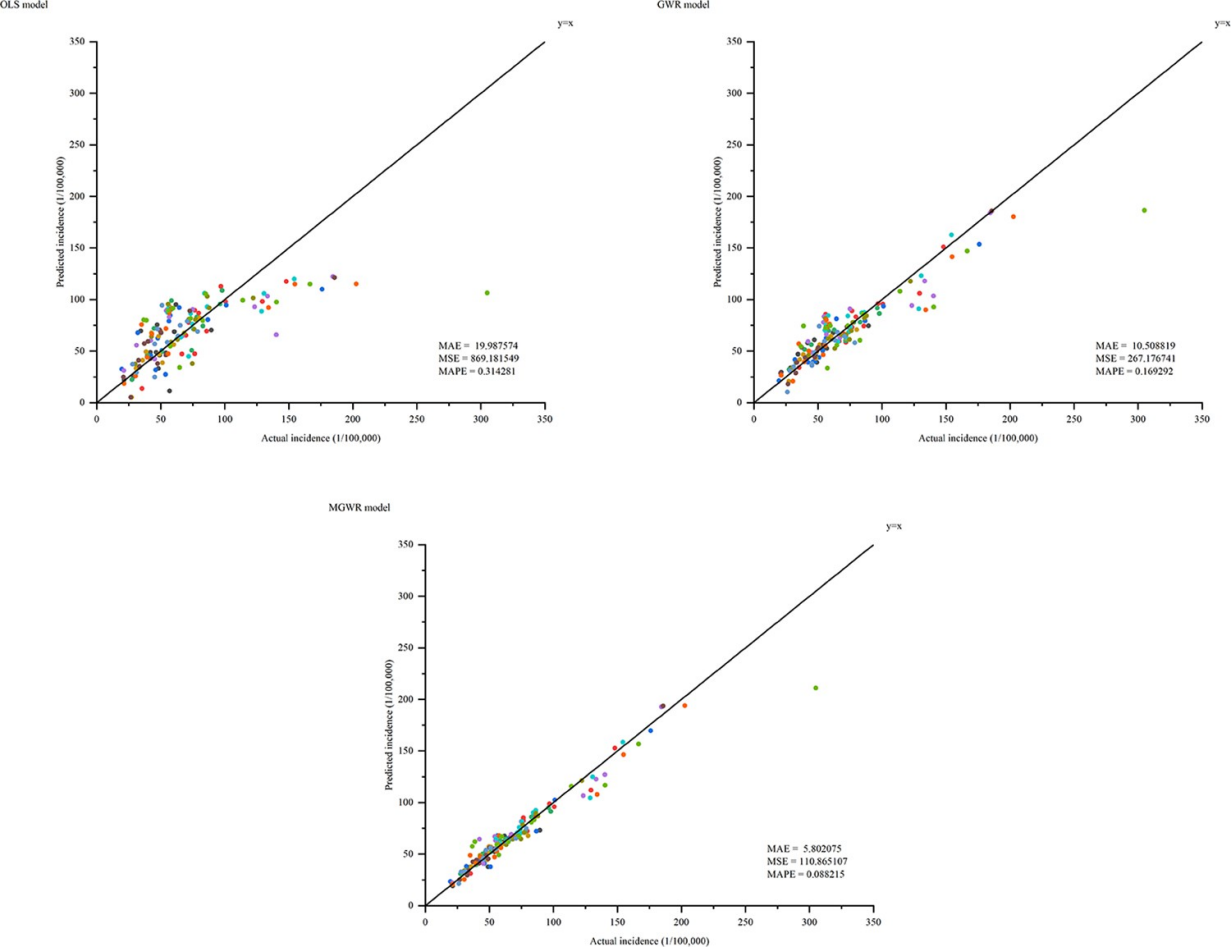
Model comparison results.

As shown in Fig 6, the MAE values of OLS, GWR and MGWR models are 19.987574, 10.508819, 5.802075, the MSE values are 869.181549, 267.176741, 110.865107 and the MAPE values are 0.314281, 0.169292 and 0.088215, respectively. The MAE, MSE and MAPE of MGWR model are better than the other two models, which are closer to the actual incidence. As shown in Table 8, the MGWR model has a smaller AIC value and residual sum of squares than the other two models. Among them, the R^2^ and the adjusted R^2^ are 0.932 and 0.910, respectively, indicating that the fitting effect of the MGWR model is better than the other two models.

**Table 8:** Comparison of model fitting indicators.

indicator	OLS	GWR	MGWR
**R** ^**2**^	0.466	0.836	0.932
**Adjust R** ^**2**^	0.429	0.797	0.910
**Residual sum of squares**	134723	41412	17184
**AIC**	1512.84	1360.13	1198.89

## 4. Results and discussion of MGWR analysis

As COVID-19 swept the world in 2019, each country struggled to deal with patients infected with COVID-19, which occupied most of medical resources. At the same time, some patients with TB failed to go to the hospital in time, resulting in a sharp drop in the number of TB cases. In order to ensure the objectivity and reliability of the analyzed data, this period is excluded in this study. This paper explores the temporal and spatial distribution of TB incidence in mainland China in the five years before the outbreak of COVID-19, and analyzes the degree of impact on TB incidence from three levels: air quality, social life, and medical level. The quantitative contribution of each influencing factor is calculated through the MGWR model, which provides a decision-making reference for the prevention and treatment of TB in other countries.

As shown in Fig 4, the areas with high incidence are mainly concentrated in the Southwestern China and the Central of China from 2014 to 2018, while the incidence of TB in East China is relatively low. The overall incidence shows a trend of high in the west and low in the east [50], with large local differences and obvious aggregation distribution in some areas. For example, the incidence in the surrounding areas of Qinghai and Guizhou is generally high and shows a positive aggregation distribution, while the incidence in the surrounding areas of Beijing and other places is low, showing a reverse aggregation distribution. Relatively backward economic development, and insufficient prevention and control of infectious diseases are the main reasons for high incidence of TB in Qinghai, Tibet, Xinjiang [18, 51, 52].

**Fig 7:**
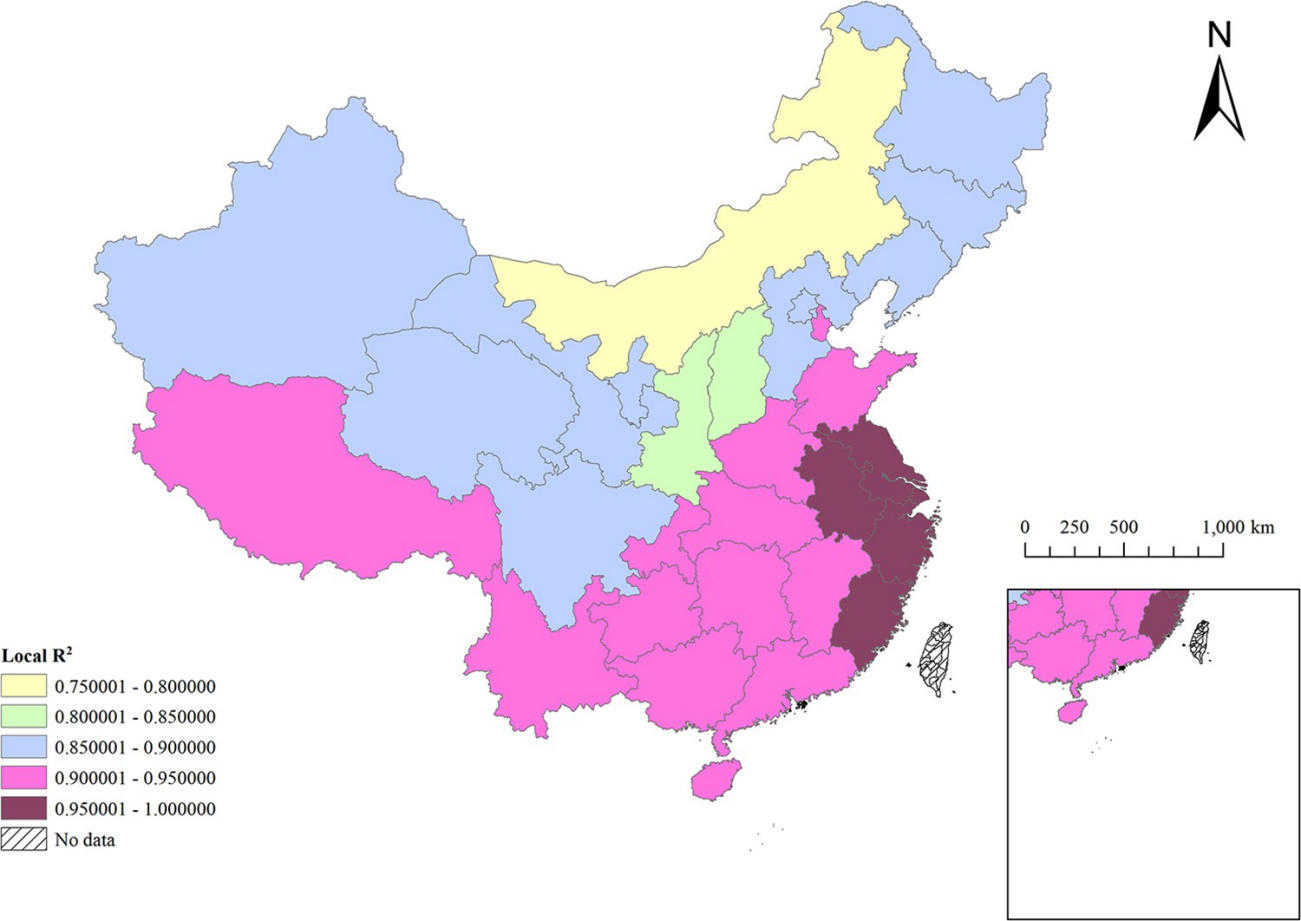
Spatial distribution of local R^2^ of MGWR model.

For the MGWR model in this study, the local R^2^ in Fig 7 is the goodness of fit of the selected influencing factors to TB incidence in each region. The results show that the local R^2^ fitting effect of each region is good, including 96.8% of the study areas with a goodness of fit exceeding 0.80, 58.1% of the study areas with a goodness of fit exceeding 0.90, and 16.1% of the study areas with a goodness of fit exceeding 0.95, which prove that the selected influencing factors have sufficient explanatory power for TB incidence. The larger R^2^ in East China and the Central of China indicates that the selected influencing factors have the best explanatory power in this area. However, dry climate, sparse vegetation, and excessive dust lead to an increase in TB [53, 54], which makes the model fit very low in North China.

**Fig 8:**
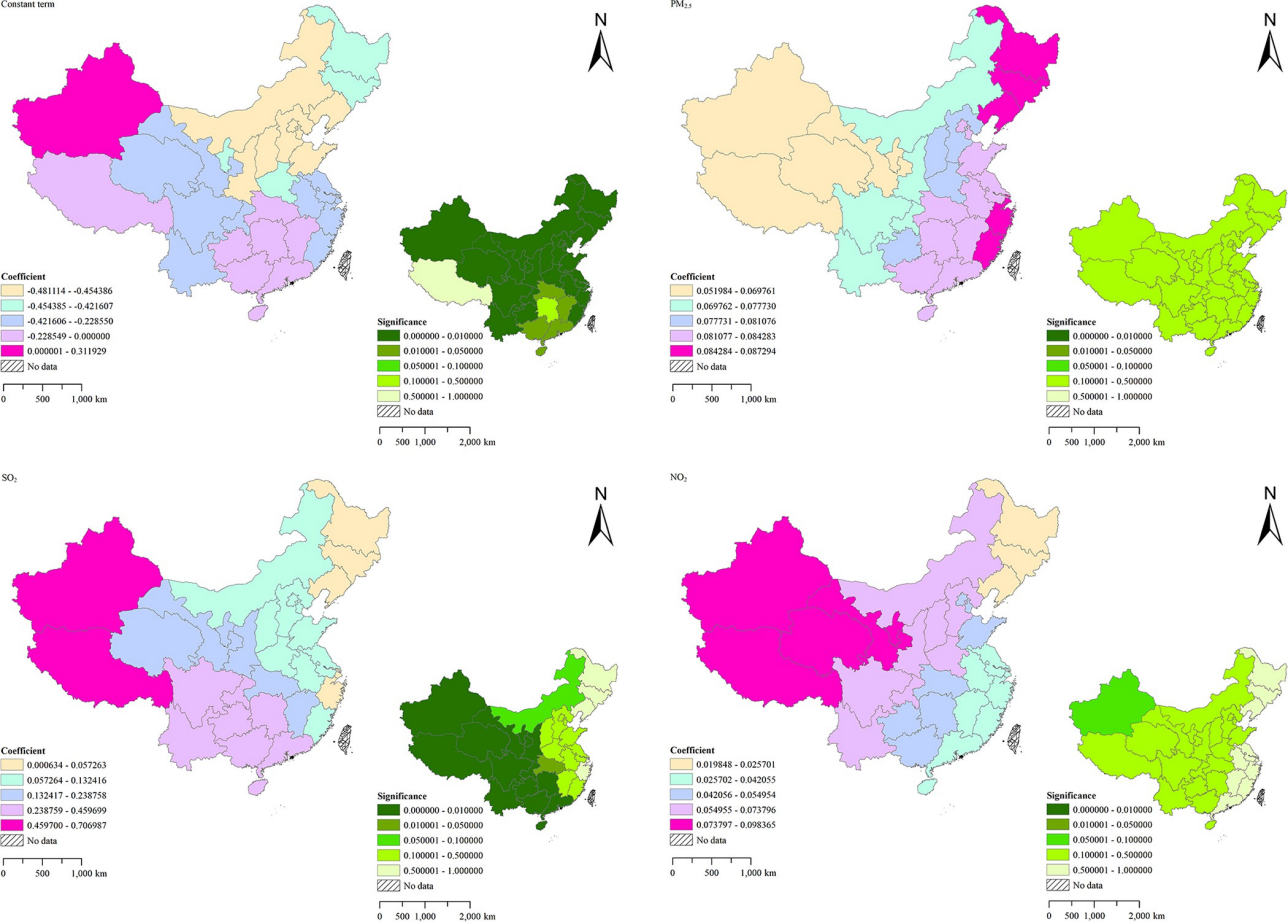
Spatial distribution of regression coefficients and significance of TB in mainland China (constant term and air quality factors).

TB is a respiratory infectious disease, and air quality directly affects its incidence [55–57]. The regression coefficients and significance of constant terms and air quality factors of the MGWR model constructed in this paper are shown in Fig 8. Among them, the trend of the coefficient of constant term is basically consistent with the trend of TB incidence in mainland China from 2014 to 2018. The eastern regions of China all show a downward trend in different degrees, while the western regions also have a small upward trend. Among the selected air quality factors, PM_2.5_ shows a significant positive correlation trend to TB incidence, and the correlation degree gradually increases from west to east. As a particle with a diameter of less than 2.5 microns, it can directly enter the lungs through respiratory tract and affect human health [58], leading to an increase in TB incidence. Moreover, SO_2_ shows a significant positive correlation trend to TB incidence, and the impact on the western regions is greater. As a colorless and irritating gas, SO_2_ has an irritating effect on the respiratory tract, and long-term exposure to high concentrations may cause lung damage, thereby increasing the possibility of TB [55, 56]. An increase in the concentration of NO_2_ also leads to the same problem. According to the research, long-term exposure to high concentrations of NO_2_ will lead to alveolar atrophy, pulmonary edema and other problems [59], which leads to the onset of TB. The regression coefficient of NO_2_ obtained by the MGWR model also proves this view. Although the absolute value of the regression coefficient is relatively low, the problem of NO_2_ emission can’t be ignored. Every country should still pay attention to this problem and achieve effective prevention and control of TB.

**Fig 9:**
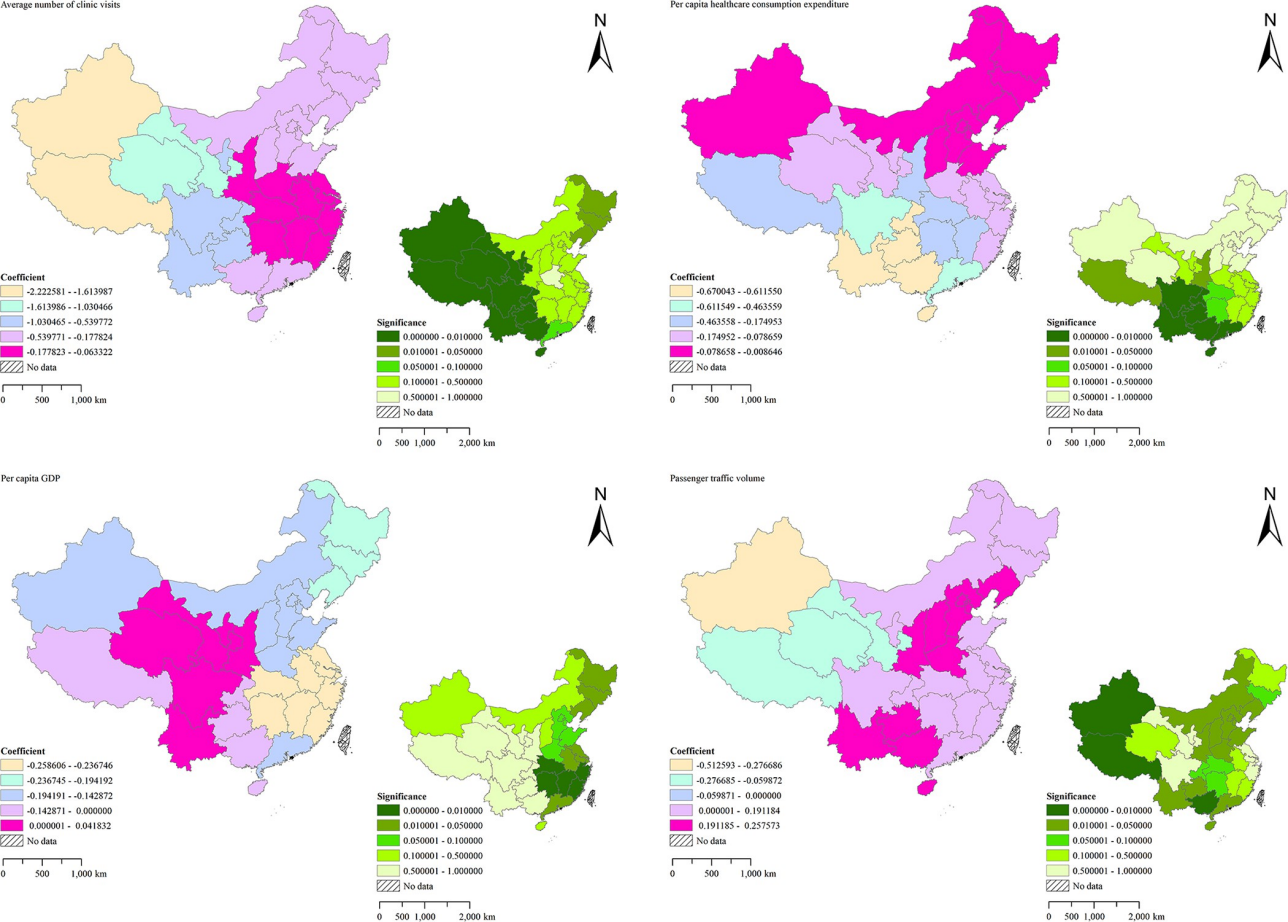
Spatial distribution of regression coefficients and significance of TB in mainland China (social life factors).

Air quality is not the only aspect that affects the spread of TB, social living standard also have a great impact on TB from many different levels [10, 11, 60]. The regression coefficients and significance of social life factors are shown in Fig 9. Among them, Average number of clinic visits has a significant negative correlation trend with TB incidence. Due to the relatively low awareness of prevention, the influence of this factor is more obvious in the western regions of China, and the confidence is generally high. Early diagnosis and timely treatment are important methods to effectively control TB, and delay diagnosis may increase infectivity and worsen the condition [61]. Therefore, especially under COVID-19, regular hospital check-ups are important for the prevention of TB. In addition, Per capita healthcare consumption expenditure and TB incidence also show a significant negative correlation trend, which have a greater impact on the Southern China and Southwestern China, and the confidence is generally high. Due to the high medical expenses of TB patients [62], it is important to appropriately increase the investment in healthcare costs during the early prevention period for the prevention of TB. However, the influence of Per capita GDP on TB incidence is polarized. Some central regions of China have a significant positive correlation trend, accounting for 19.35% of the study areas. According to the research, people in economically underdeveloped areas are more likely to suffer from TB [1, 18, 29], because these areas often overemphasize regional development but neglect the control of TB. Aiming at the problems in these areas, the suggestion put forward in this paper is that these areas should focus on the development of economic construction and disease control at the same time. In other regions of China, Per capita GDP and the incidence of TB show a negative correlation trend, accounting for 80.65% of the study areas. With the development of regional economy, these regions have strengthened the control of TB, providing an example for other countries that economic growth drives disease prevention. The effect of Passenger traffic volume on TB incidence also shows a significant polarization phenomenon, and the positive correlation trend is significant in the North China, Northeastern China, the Central of China, Southern China and East China, accounting for 87.10% of the study areas. Due to the relatively developed economy, the improvement of transportation convenience, and the continuous increase in the migration of floating population, the possibility of contacting other TB patients on the transport has also increased, resulting in an upward trend in the incidence of TB in these regions [63]. However, the negative correlation trend between Passenger traffic volume and the incidence of TB in the Northeastern China and Southwestern China, accounting for 12.90% of the study areas. Compared with other areas, these areas have lower population density and weaker economic strength, resulting in the migration of indigenous people to economically developed regions, such as the eastern regions of China. With the improvement of traffic convenience, the population flow in the western regions of China is more obvious. The indigenous people in the western regions of China migrate to other regions, resulting to a downward trend in the incidence of TB, while an upward trend in other regions, which also explains the phenomenon why the eastern regions of China is positively correlated.

**Fig 10:**
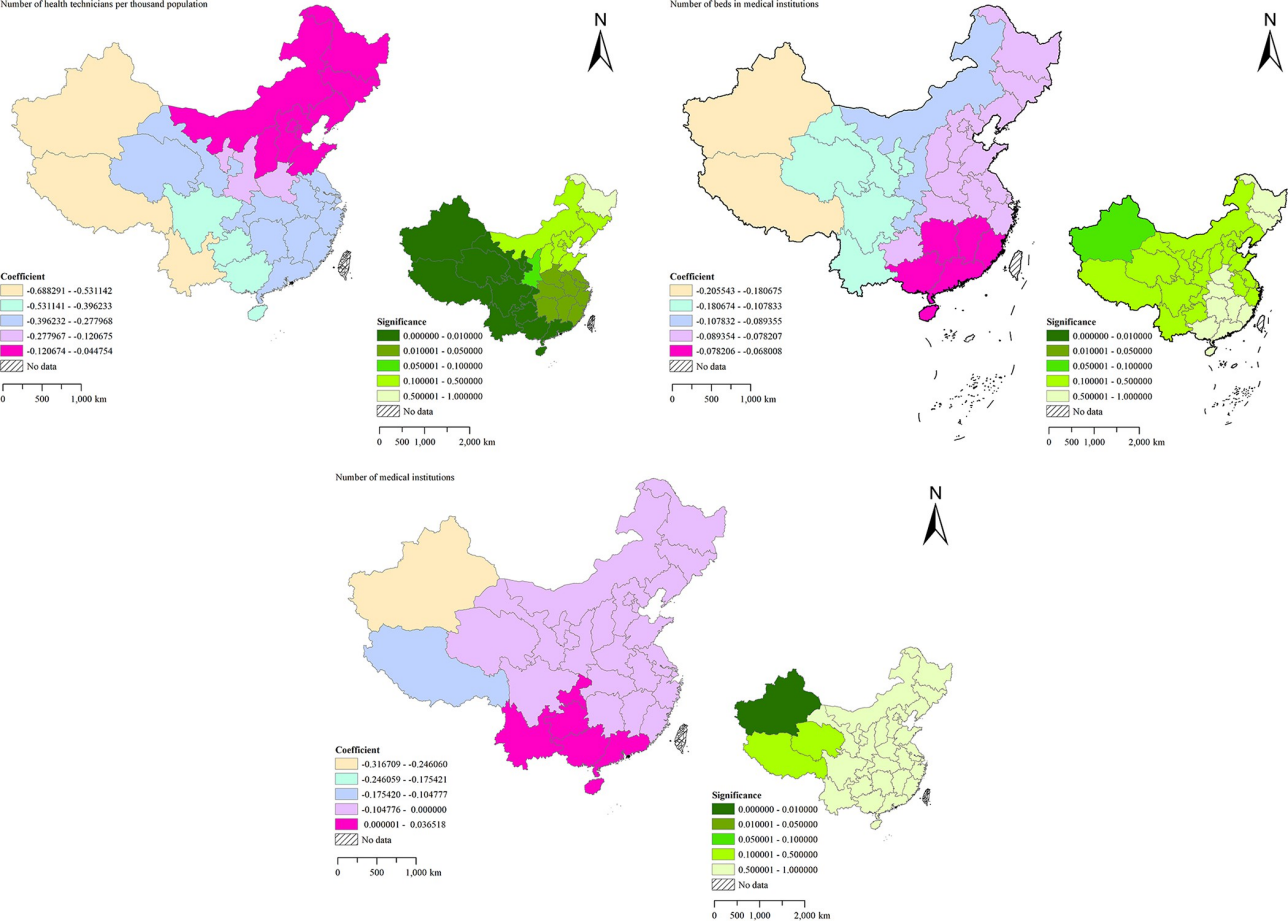
Spatial distribution of regression coefficients and significance of TB in mainland China (medical level factors).

The medical level is also crucial to the prevention and treatment of TB [64]. The regression coefficients and significance of medical level factors are shown in Fig 10. Among them, Number of health technicians per thousand population and TB incidence show a significant negative correlation trend. The Southwestern China and Northwestern China are significantly affected by this factor, and the overall confidence in these regions is relatively high, which effectively proves the importance of this factor to the prevention and control of TB in these regions. Therefore, an appropriate increase in medical technicians can effectively reduce the incidence of TB [51]. Furthermore, Number of beds in medical institutions and TB incidence also show a significant negative correlation trend, which has a greater impact on the Northwestern China and Southwestern China. With the gradual increase in hospital investment and the number of beds, the situation that once shortened the hospitalization days of TB patients no longer appeared, resulting in more and more effective treatment of TB today [61]. Although the absolute value of the overall regression coefficient is low, appropriately increasing the number of beds in medical institutions can still effectively reduce the number of TB patients. However, the impact of Number of medical institutions on the incidence of TB is polarized. This factor has a significant positive correlation trend with TB in Southern China, accounting for 19.35% of the study areas. Due to the dense population and extremely fast population growth rate in these areas, there are also more medical institutions in these areas, but these reasons also make it more difficult to control the spread of TB. The number of medical institutions in other regions of China has a significant negative correlation trend with TB incidence, accounting for 80.65% of the study areas. Abundant medical resources enable the control of TB to be well managed [65]. Therefore, targeted increase of medical institutions can more effectively prevent the spread of TB.

## 5. Conclusion

Due to the decline in the authenticity and reliability of TB under the COVID-19, this paper uses spatial autocorrelation methods to analyze the spatial distribution of TB incidence in mainland China from 2014 to 2018, and introduces MGWR model to analyze its spatial heterogeneity. Using the collected data on air quality, social life and medical level, the potential factors affecting the transmission of TB are analyzed. Moreover, through the comparative experiment of OLS model and GWR model, the superiority of MGWR model in dealing with such problems is verified, and it provides decision-making references for China and other countries in the prevention and control of TB under the impact of major infectious diseases such as COVID-19. The following are the main findings of this study:

1. The overall incidence of TB in mainland China shows a downward trend from 2014 to 2018, and the distribution of TB cases in the study areas shows spatial aggregation and spatial heterogeneity during this period.
2. Among the air quality factors, PM_2.5_, SO_2_ and NO_2_ are positively correlated with TB incidence. Among the social life factors, Average number of clinic visits, Per capita healthcare consumption expenditure, Per capita GDP and Passenger traffic volume are generally negatively correlated with TB incidence. Among the medical level factors, Number of health technicians per thousand population, Number of beds in medical institutions and Number of medical institutions generally have a negative effect. Therefore, improving air quality, promoting national economic development, and increasing investment in medical resources can effectively control the spread of TB.
3. The MGWR model introduces spatial position characteristics of each sample point, and divides them into global variables and local variables according to the effect of each influencing factor on the model. Therefore, it makes the influencing factor have better spatial-temporal analysis ability, and its overall fitting effect is significantly better than OLS model and GWR model.

However, this paper also has some limitations. In terms of data, if we can collect real and reliable data on the incidence of TB under the COVID-19 and compare it with this paper, we can provide more reliable reference opinions for the prevention and control of TB, and there may still be some influencing factors that have not been put into the model for discussion, resulting in a reduction in the explanatory power of the model. Follow-up research can further add other influencing factors and research data to make the model more perfect.

## Supporting information

S1 Dataset TB data and selected influencing factors data from 2014 to 2018. (XLSX)

S2 Dataset Monthly incidence data of TB in 2014–2018. (XLSX)

S3 Dataset MGWR coefficient table. (XLSX)

S4 Dataset GWR coefficient table. (XLSX)
